# Supplementary material for: Eye movements during free viewing to maximize scene understanding
Source: Nat Commun. 2025 Dec 21;17:940. doi: 10.1038/s41467-025-67673-w (PMC12830957; doi:10.1038/s41467-025-67673-w)
Supplement: Supplementary file 1 — Supplementary Information [file 41467_2025_67673_MOESM1_ESM.pdf]

Supplementary Materials for

Eye Movements During Free Viewing to Maximize Scene Understanding

Shravan Murlidaran and Miguel P. Eckstein

*Lead Contact. Email: smurlidaran@ucsb.edu

This PDF file includes:

Figs. S1 to S7

Figure. S1.

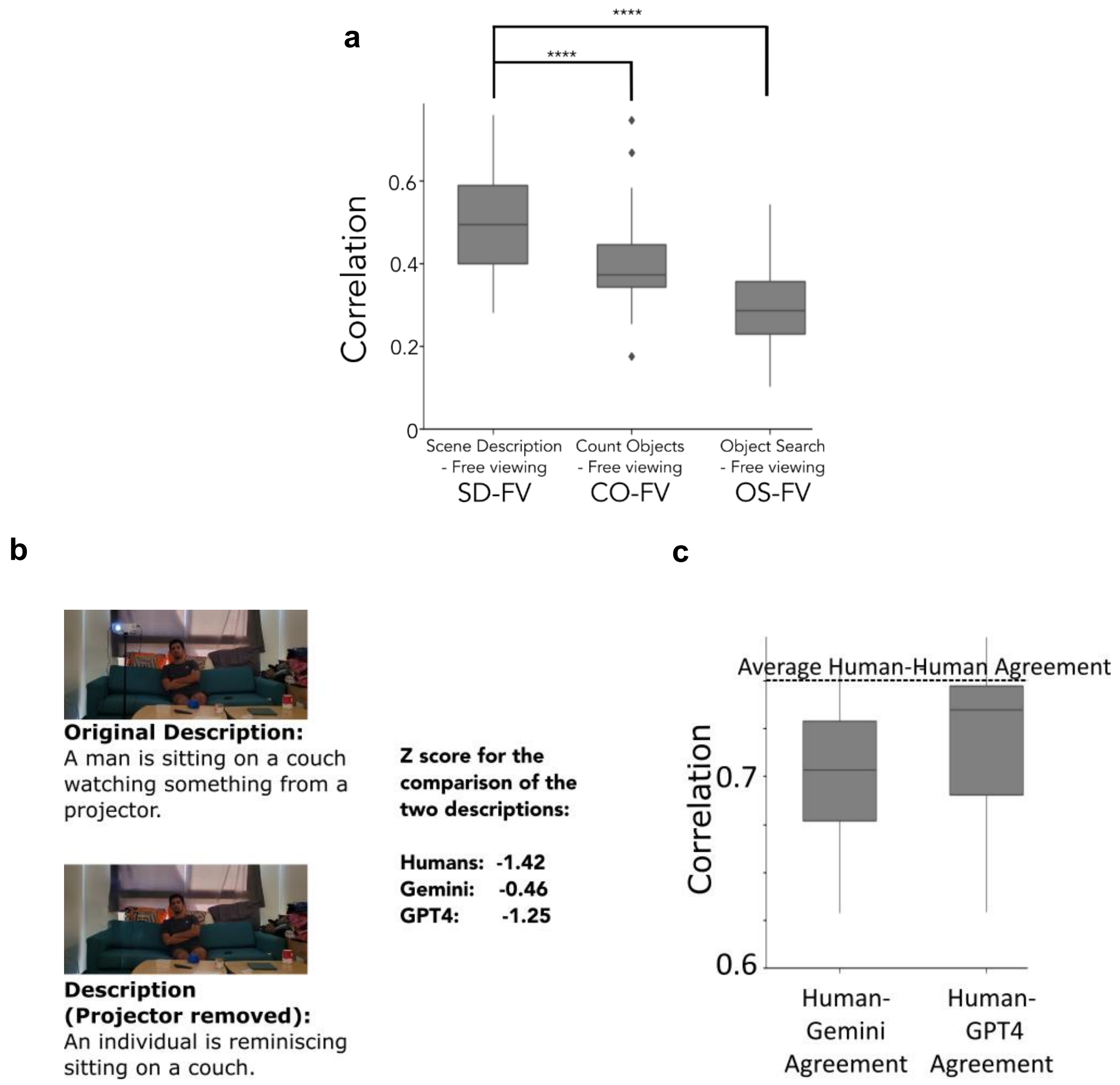

(a) Correlation across fixation heatmaps (n=25 observers) for different conditions (scene description, count objects, object search and free viewing). We observed similar results to those of the analysis with groups of 12 observers per condition reported in the main text (Figure 2c in the main text). **(b)** A sentence description for the original image (top) and for the image with the projector removed (bottom). To the right, example of the scores obtained from the LLM and human ratings for the similarity of two image descriptions. **(c)** The average correlation of individual human raters with semantic cosine similarity using GPT4 language embeddings and Gemini embeddings is comparable to the average inter-human rating correlation (dashed horizontal line). The above comparison used the human ratings (18 raters) and LLM embeddings obtained from the object removal experiment (averaged across all images). **** ($p < 0.0001$) A one-tailed bootstrapped analysis was conducted to test the significance of all the results. In **(c)**, the line within the box indicates the median. The box spans the interquartile range (IQR), and the whiskers extend to the most extreme values within 1.5×IQR across participants.

Figure. S2.

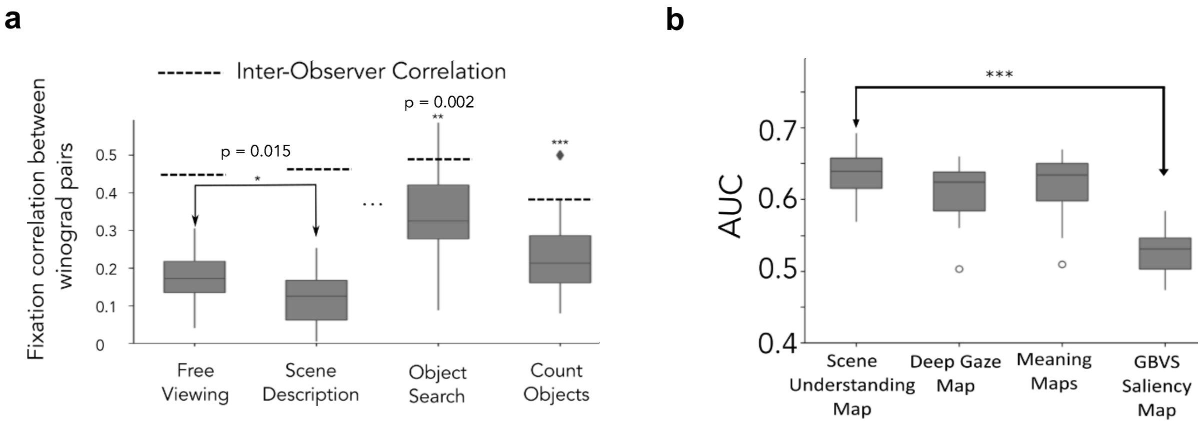

(a) Fixation heatmap (n=12 observers for each condition) correlation across Winograd pairs. The dotted lines indicate the same image inter-observer fixation heatmap correlation (12 vs. 12 observer heatmaps) averaged across the images. These results show that the difference between the same image inter-observer and the Winograd image-pair correlations is significantly higher for the scene description condition than for free viewing. The correlation difference observed for object search and counting objects is significantly lower when compared to free-viewing. **(b)** A shuffled AUROC analysis revealed that the SUM maps have the highest performance in predicting human fixations (25 participants per image, 36 images) during free viewing (AUC = 0.64) and was significantly different from GBVS maps (AUC = 0.53, bootstrap, $p < 0.001$) but was not significantly different from meaning maps (AUC = 0.62, bootstrap, $p = 0.18$) or DeepGaze (AUC = 0.61, bootstrap, $p = 0.94$). (*** = $p < 0.001$; ** = $p < 0.01$; * $p < 0.05$). A one-tailed bootstrapped analysis was conducted to test the significance of all the results. In both plots, the line within the box indicates the median. The box spans the interquartile range (IQR), and the whiskers extend to the most extreme values within 1.5×IQR across images for **(a)** and across participants for **(b)**.

Figure. S3.

a

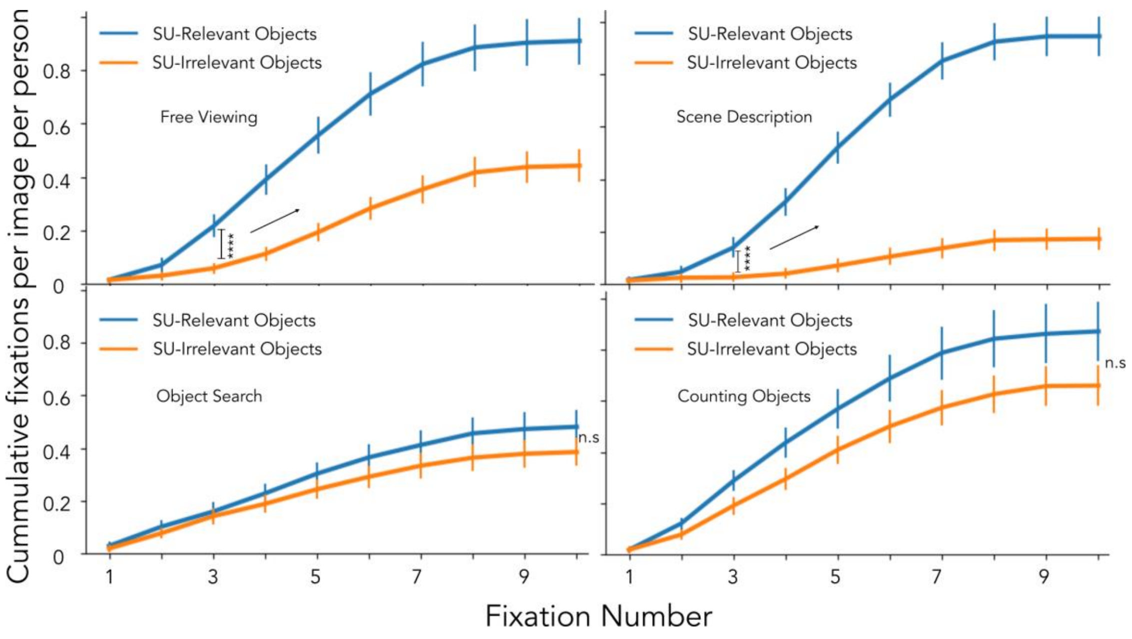
Cummulative fixations per image per person
SU-Relevant Objects
SU-Irrelevant Objects
Free Viewing
Scene Description
Object Search
Counting Objects
n.s
0
0.2
0.4
0.6
0.8
1
3
5
7
9
Fixation Number

b

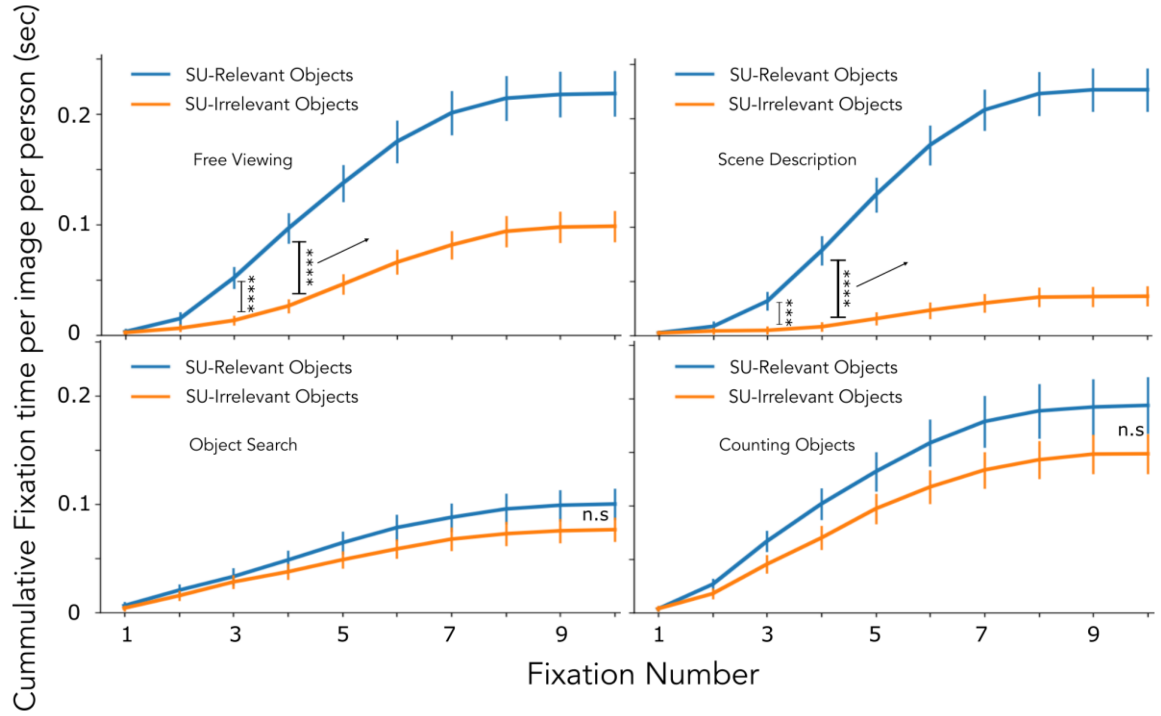
Cummulative Fixation time per image per person (sec)
SU-Relevant Objects
SU-Irrelevant Objects
Free Viewing
Scene Description
Object Search
Counting Objects
n.s
0
0.1
0.2
1
3
5
7
9
Fixation Number

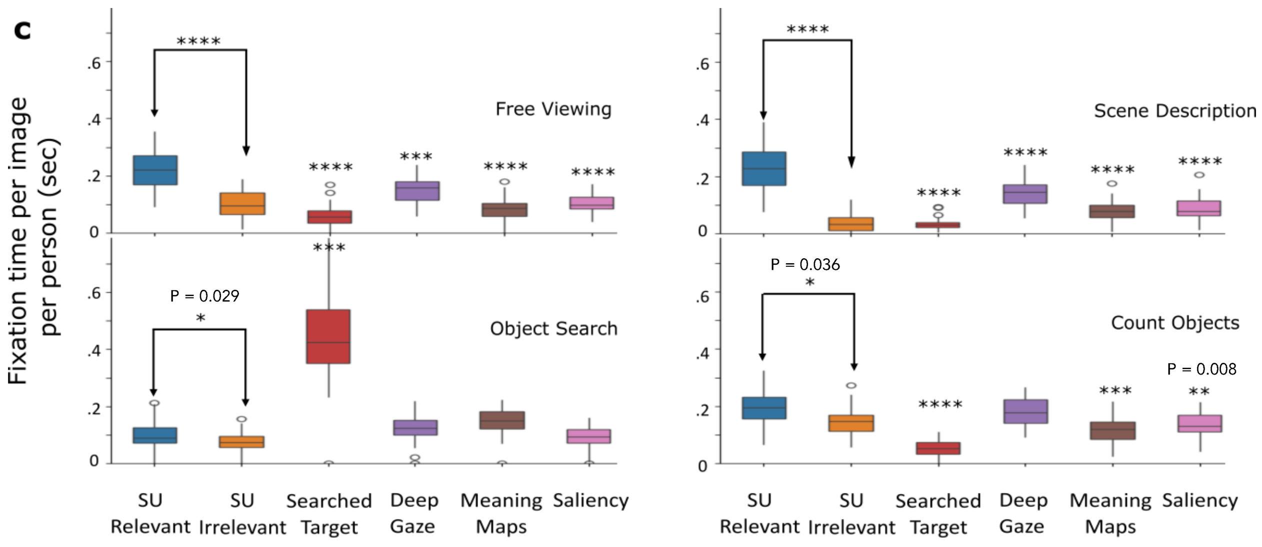

(a) Cumulative fixation frequency for SU-relevant and SU-irrelevant object categories shows a significant difference in free-viewing and scene description conditions from the 3rd fixation onwards. There is a trend of higher fixation frequency for SU-relevant vs. SU-irrelevant objects in search and counting-object conditions, but did not reach statistical at any fixation measured (until the 10th). **(b)** Cumulative time-weighted fixations for SU-relevant and SU-irrelevant objects show similar result patterns for all four conditions. **(c)** Time-weighted fixation for object categories. This measure also shows the same trend as fixation frequency. Observers fixated longer on SU-relevant vs. SU-irrelevant objects. 25 participants per image, 36 images in all analysis. (**** = p < 0 .0001; *** = p < 0.001; ** = p < 0.01; * p < 0.05). A one-tailed bootstrapped analysis was conducted to test the significance of all the results. Each analyses had their significance levels corrected for the False Discovery Rate (α=0.05, 40 comparisons each in **(a)** and **(b)**, 20 comparisons in **(c)**). In **(a)** and **(b)**, the central measure is the mean and the error bars show the 68% bootstrap confidence interval. In **(c)**, the line within the box indicates the median. The box spans the interquartile range (IQR), and the whiskers extend to the most extreme values within 1.5×IQR across participants.

Figure S4.

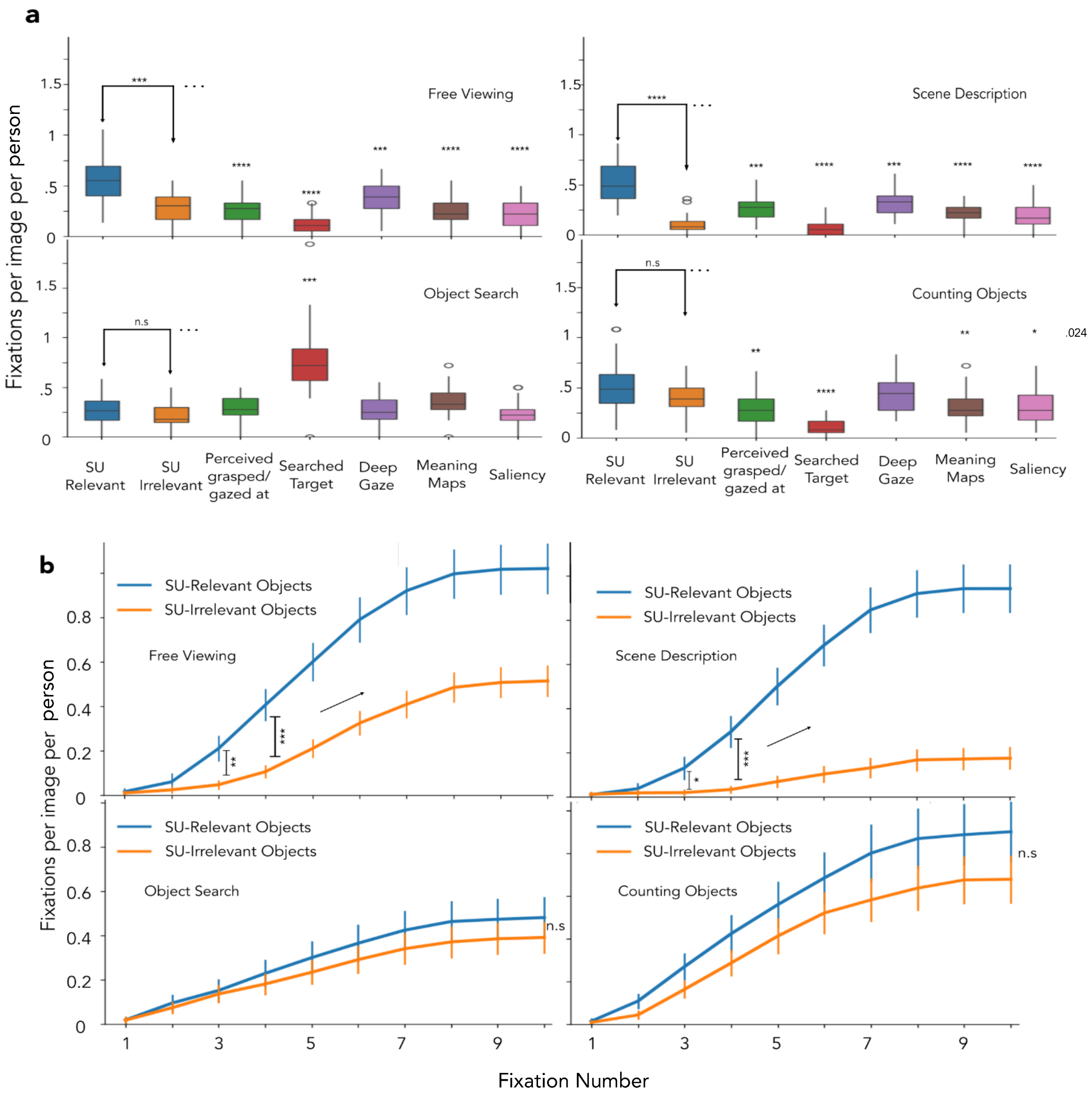

(a) Scenes with "No gaze" (scenes for which SU-relevant objects are different than objects judged to be grasped or gazed at) show similar pattern of effects in free viewing and scene description conditions across all object categories. However, the difference in fixation frequency on SU-relevant and SU-irrelevant objects is no longer significant in the object search and counting objects conditions. **(b)** Cumulative fixation frequency shows that the difference between the fixation frequency on SU-relevant and SU-irrelevant categories is significant from the 3rd fixation onwards for scene description and free viewing conditions, and no significant difference was found for the other two conditions at least until the 10th fixation. 25 participants per image, 20 images in all analysis. (**** = $p < 0.0001$; *** = $p < 0.001$; ** = $p < 0.01$; * $p < 0.05$). A one-tailed bootstrapped analysis was conducted to test the significance of all the results. Each analyses had their significance levels corrected for the False Discovery Rate (α=0.05, 24 comparisons in **(a)**, 40 comparisons in **(b)**). In **(b)**, the line within the box indicates the median. The box spans the interquartile range (IQR), and the whiskers extend to the most extreme values within 1.5×IQR across participants. In **(a)**, the central measure is the mean, and the error bars show the 68% bootstrap confidence interval.

Figure S5.

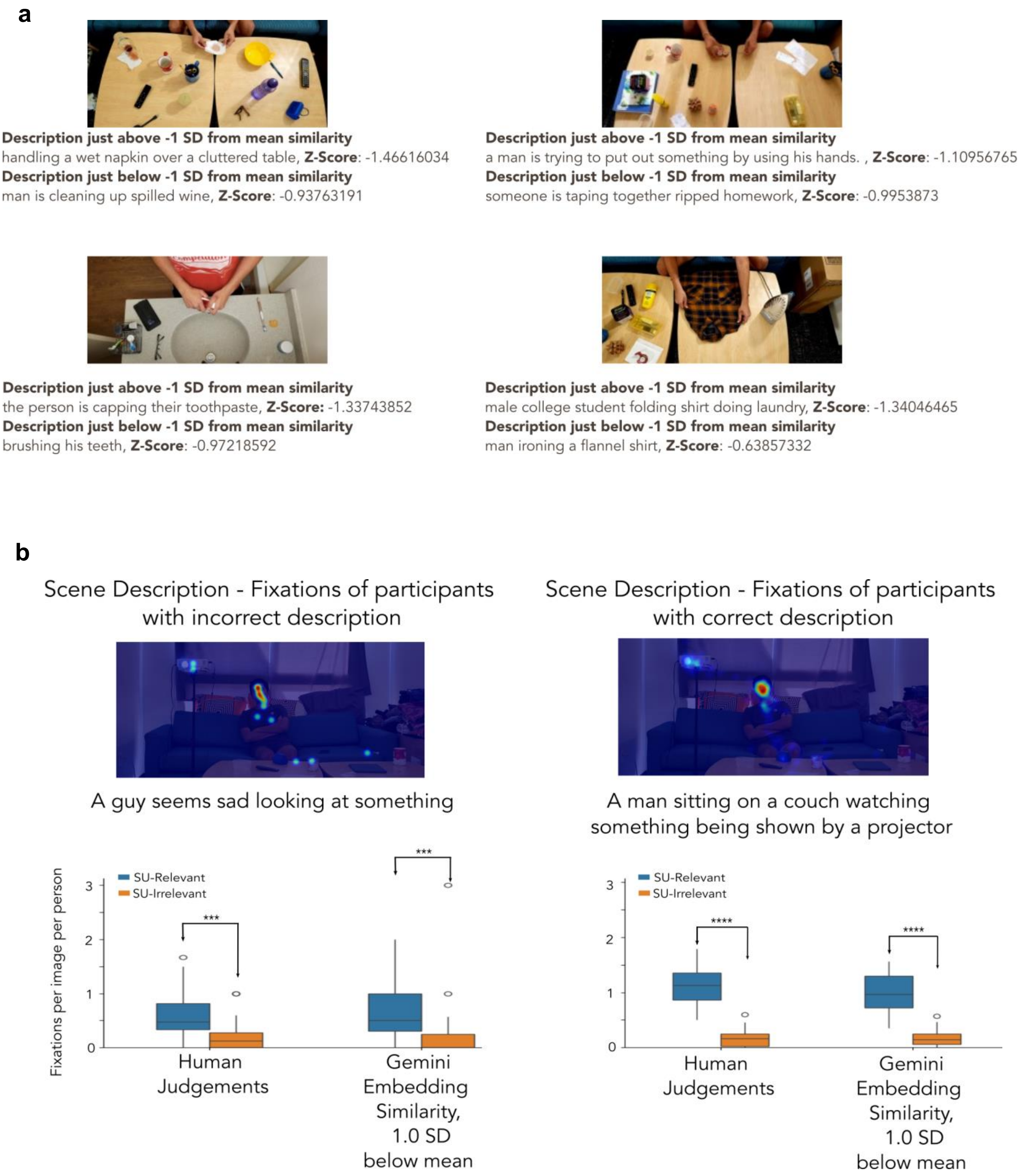
a
Description just above -1 SD from mean similarity
handling a wet napkin over a cluttered table, Z-Score: -1.46616034
Description just below -1 SD from mean similarity
man is cleaning up spilled wine, Z-Score: -0.93763191
Description just above -1 SD from mean similarity
a man is trying to put out something by using his hands. , Z-Score: -1.10956765
Description just below -1 SD from mean similarity
someone is taping together ripped homework, Z-Score: -0.9953873
Description just above -1 SD from mean similarity
the person is capping their toothpaste, Z-Score: -1.33743852
Description just below -1 SD from mean similarity
brushing his teeth, Z-Score: -0.97218592
Description just above -1 SD from mean similarity
male college student folding shirt doing laundry, Z-Score: -1.34046465
Description just below -1 SD from mean similarity
man ironing a flannel shirt, Z-Score: -0.63857332
b
Scene Description - Fixations of participants with incorrect description
A guy seems sad looking at something
Scene Description - Fixations of participants with correct description
A man sitting on a couch watching something being shown by a projector
SU-Relevant
SU-Irrelevant
Fixations per image per person
0
1
2
3
***
***
****
****
Human Judgements
Gemini Embedding Similarity, 1.0 SD below mean

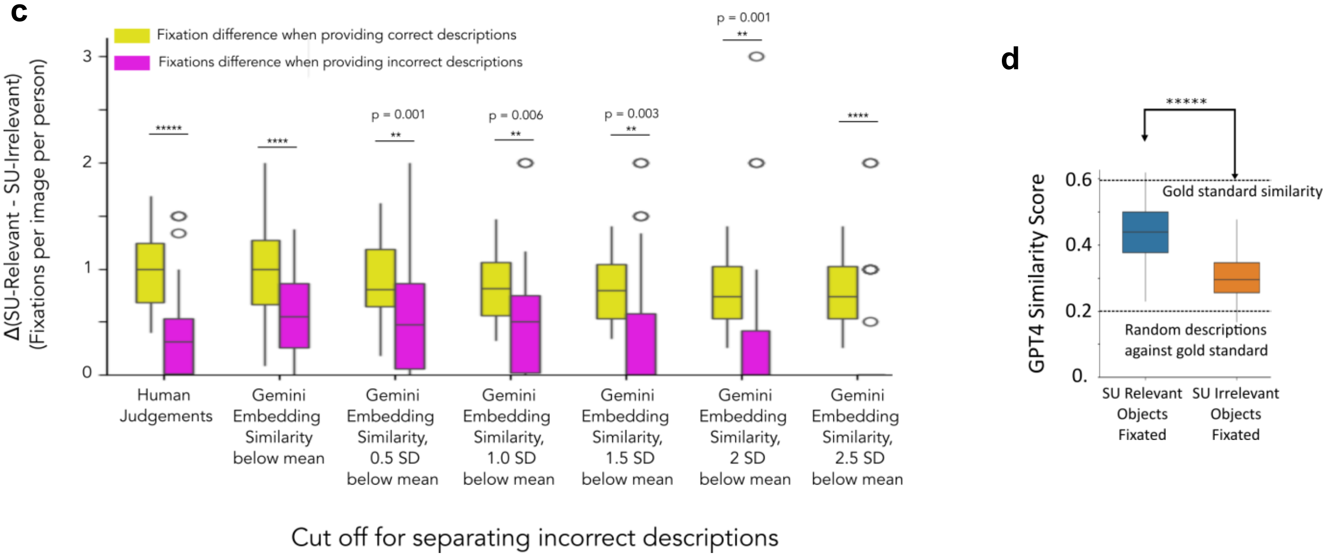

(a) Examples of descriptions with z-scores below and above -1 SD (threshold) from the mean description similarity rating provided by participants during the scene description condition for each image. Descriptions for all images near the decision threshold of -1 SD are available in Mendeley[1]. **(b)** (Top) Example Winograd image pair with the fixations divided across groups of observers that correctly and incorrectly described images (see methods for the procedure to classify a description as correct or incorrect). (Bottom) Distribution of fixations on object categories in the scene description condition. Participants tend to fixate on the SU-relevant objects more frequently when accurately describing the scene. This result holds irrespective of whether one uses human judgments to determine whether the participants' image descriptions were correct/incorrect (relative to the gold standard) or uses the LLM embedding similarity measure (1.0 standard deviation, SD from the mean similarity rating). **(c)** The difference in fixations on SU-relevant and SU-irrelevant object categories split by participants who gave a correct versus an incorrect description, is shown as a function of the decision threshold for separating the incorrect and correct description sets. Images with correct descriptions, determined using various categorization criteria (varying SD from the mean cosine similarity of Gemini embeddings), always led to higher fixation frequency on SU-relevant vs. SU-irrelevant objects than images with incorrect descriptions. As SD increases, the threshold for what is considered an incorrect description increases, resulting in fewer data samples for the incorrect description category (pink bars). The fixation difference across SU-relevant and SU-irrelevant objects was significantly larger for correct vs. incorrect descriptions. This result generalized across all SD decision thresholds (for the LLM embedding similarity measure) and for the human correct/incorrect classification judgments. **(d)** The average similarity of descriptions from participants fixating at the SU-relevant and SU-irrelevant locations (15 participants per fixation location, 36 images) compared to the ground truth image descriptions, quantified using GPT4 embeddings to compute cosine similarity, shows similar results as Gemini embeddings, related to Figure 7. For all graphs: ***** = $p < 0.00001$; *** = $p < 0.001$; ** = $p < 0.01$. A one-tailed bootstrapped analysis was conducted to test the significance of all the results. In all figures, the line within the box indicates the median. The box spans the interquartile range (IQR), and the whiskers extend to the most extreme values within 1.5×IQR across participants for **(b)** and **(c)** and across images for **(d)**.

Figure S6.

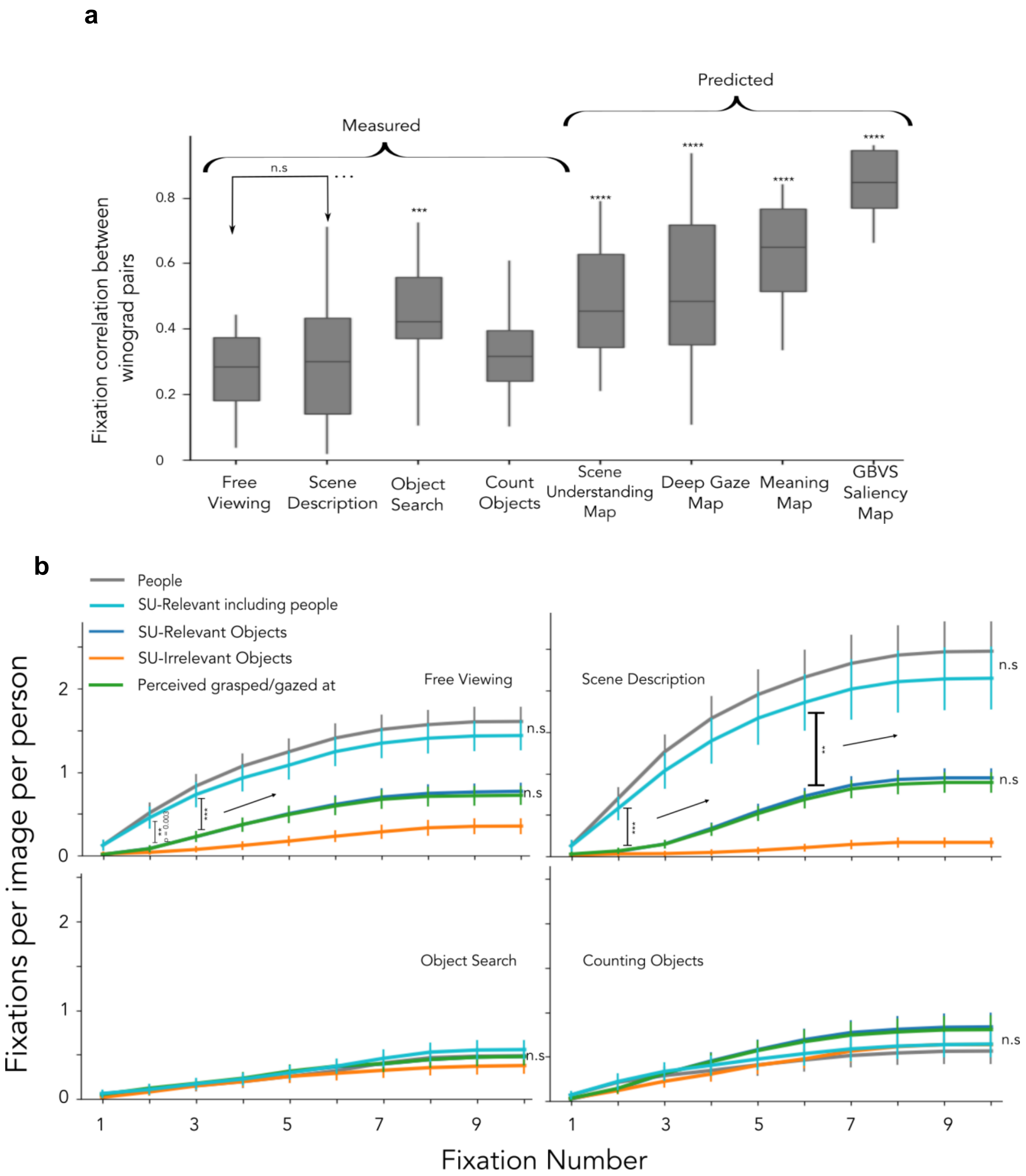
a
Predicted
Measured
n.s
Fixation correlation between winograd pairs
0.8
0.6
0.4
0.2
0
***
****
****
****
****
Free Viewing
Scene Description
Object Search
Count Objects
Scene Understanding Map
Deep Gaze Map
Meaning Map
GBVS Saliency Map
b
People
SU-Relevant including people
SU-Relevant Objects
SU-Irrelevant Objects
Perceived grasped/gazed at
Free Viewing
Scene Description
Object Search
Counting Objects
n.s
Fixations per image per person
Fixation Number

c

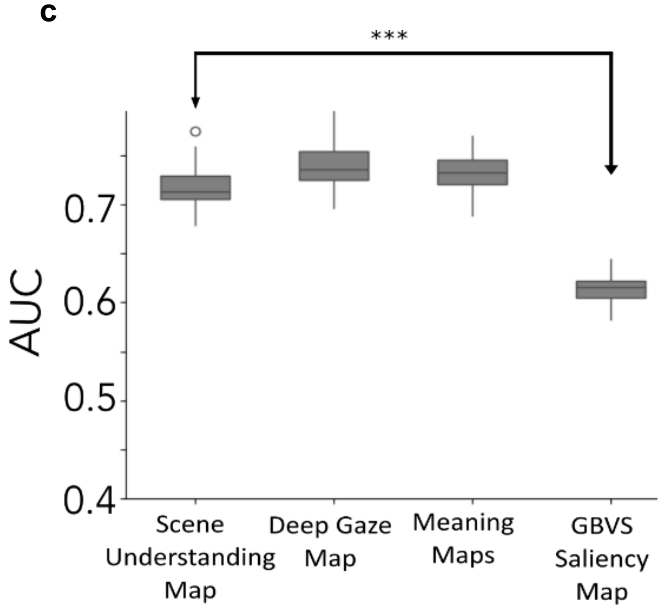

(a) Fixation heatmap correlation across Winograd pairs with human fixations/model fixation predictions to people included in the analysis (images=36, participants=25). The Winograd correlation for free viewing and scene description increases due to the many fixations on people in those two conditions (people are at similar locations in each Winograd pair). With the inclusion of fixations on people, the heatmap correlation across Winograd images for counting objects was not significantly different from that of free viewing. This also increases the SUM correlation across Winograd pairs, as the SUM predicts people to be the most important in 77% of the scenes. **(b)** Patterns similar to the main results (Figure 8d) are observed for the cumulative frequency of fixations on object categories for the image subset (images=20, participants=25), where the object perceived to be grasped/gazed at is the same as the SU-relevant object. The slight difference in SU-relevant and perceived grasped/gazed at is related to some images having more than one SU-relevant object. **(c)** A shuffled AUROC analysis for the entire image data set revealed that the SUM maps have similar performance in predicting human fixations during free viewing (AUC = 0.72) to meaning maps (AUC = 0.73, bootstrap, $p = 0.22$) or DeepGaze (AUC = 0.74, bootstrap, $p = 0.09$), and are significantly different from GBVS maps (AUC = 0.61, bootstrap, $p < 0.001$), (images=36, participants=25). (**** = $p < 0.0001$; *** = $p < 0.001$; ** = $p < 0.01$). A one-tailed bootstrapped analysis was conducted to test the significance of all the results. Analyses for comparisons in (b) had their significance levels corrected for the False Discovery Rate (α=0.05, 30 comparisons in **(a)**). In figures **(a)** and **(c)**, the line within the box indicates the median. The box spans the interquartile range (IQR), and the whiskers extend to the most extreme values within 1.5×IQR across images in **(a)** and across participants in **(c)**. In **(b)**, the central measure is the mean, and the error bars show the 68% bootstrap confidence interval.

Figure S7.

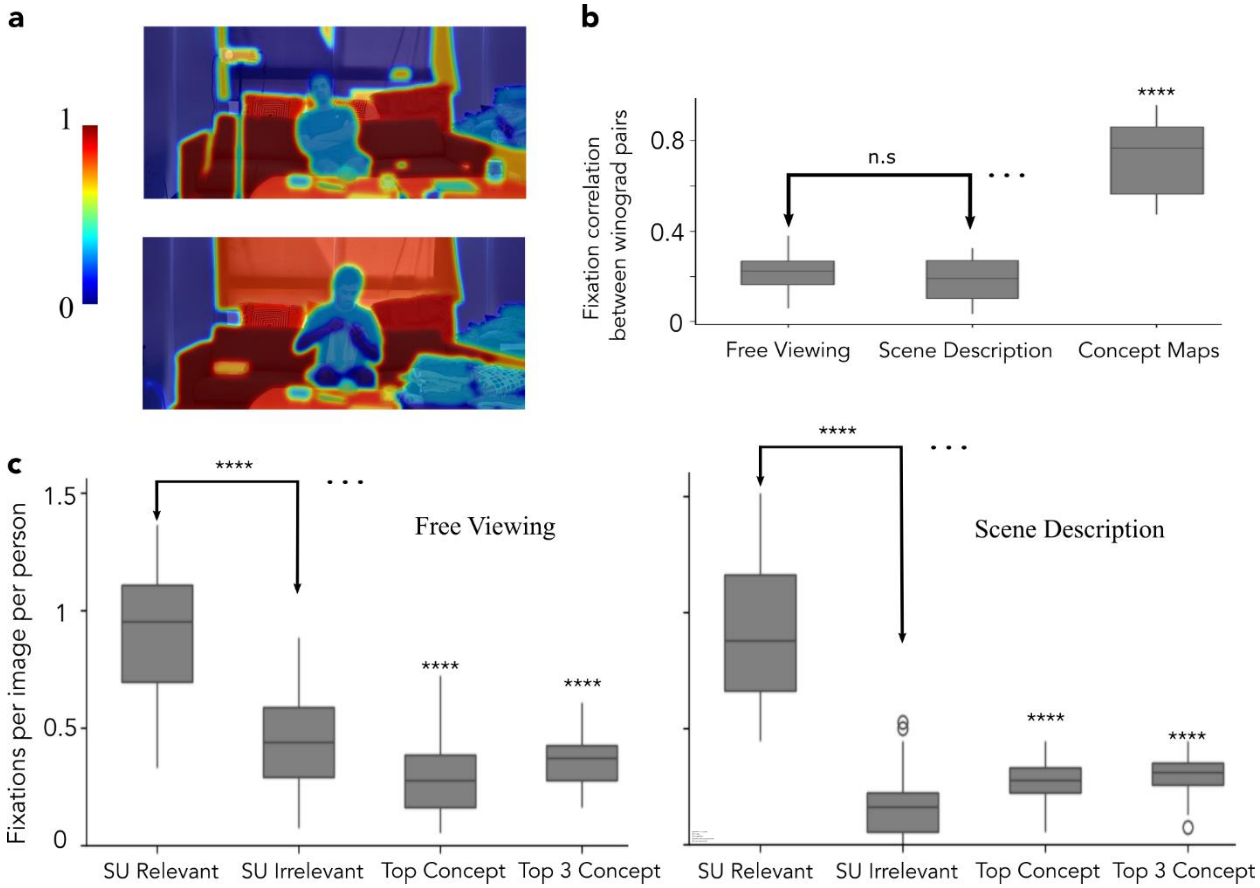

(a) Example concept maps[2] generated for a Winograd image pair. **(b)** The correlation across Winograd pairs for concept maps is significantly higher than that observed in the Winograd correlations of free viewing or scene description fixational heatmaps. This suggests that the concept maps do not vary greatly across Winograd image pairs. **(c)** The frequency of fixations on SU-relevant objects is significantly higher than the top or top 3 object predictions in the concept maps. (**** = $p < 0.0001$). A one-tailed bootstrapped analysis was conducted to test the significance of all the results. In figures **(b)** and **(c)**, the line within the box indicates the median. The box spans the interquartile range (IQR), and the whiskers extend to the most extreme values within 1.5×IQR across images in **(b)** and across participants in **(c)**.

Supplementary References:

[1] Murlidaran, Shravan; Eckstein, Miguel (2025), “Winograd Images Dataset”, Mendeley Data, V1, doi: 10.17632/z6jb259pcd.1

[2] Hayes, Taylor R., and John M. Henderson. "Looking for semantic similarity: what a vector-space model of semantics can tell us about attention in real-world scenes." *Psychological Science* 32, no. 8 (2021): 1262-1270.
