# Supplementary material for: Eye movements during free viewing to maximize scene understanding
Source: Nat Commun. 2025 Dec 21;17:940. doi: 10.1038/s41467-025-67673-w (PMC12830957; doi:10.1038/s41467-025-67673-w)
Supplement: Supplementary file 2 — Reporting Summary [file 41467_2025_67673_MOESM2_ESM.pdf]

nature portfolio

Corresponding author(s): Shravan Murlidaran

Last updated by author(s): Oct 21, 2025

Reporting Summary

Nature Portfolio wishes to improve the reproducibility of the work that we publish. This form provides structure for consistency and transparency in reporting. For further information on Nature Portfolio policies, see our Editorial Policies and the Editorial Policy Checklist.

Statistics

For all statistical analyses, confirm that the following items are present in the figure legend, table legend, main text, or Methods section.

n/a	Confirmed	
☐	☒	The exact sample size (*n*) for each experimental group/condition, given as a discrete number and unit of measurement
☐	☒	A statement on whether measurements were taken from distinct samples or whether the same sample was measured repeatedly
☐	☒	The statistical test(s) used AND whether they are one- or two-sided<br>*Only common tests should be described solely by name; describe more complex techniques in the Methods section.*
☐	☒	A description of all covariates tested
☐	☒	A description of any assumptions or corrections, such as tests of normality and adjustment for multiple comparisons
☐	☒	A full description of the statistical parameters including central tendency (e.g. means) or other basic estimates (e.g. regression coefficient) AND variation (e.g. standard deviation) or associated estimates of uncertainty (e.g. confidence intervals)
☐	☒	For null hypothesis testing, the test statistic (e.g. *F*, *t*, *r*) with confidence intervals, effect sizes, degrees of freedom and *P* value noted<br>*Give P values as exact values whenever suitable.*
☒	☐	For Bayesian analysis, information on the choice of priors and Markov chain Monte Carlo settings
☒	☐	For hierarchical and complex designs, identification of the appropriate level for tests and full reporting of outcomes
☐	☒	Estimates of effect sizes (e.g. Cohen's *d*, Pearson's *r*), indicating how they were calculated

Our web collection on statistics for biologists contains articles on many of the points above.

Software and code

Policy information about availability of computer code

Data collection	SR Eyelink research is used to collect eye movement data, Python (3.6 or above) and PsychoPy (2020 or above) are used to set up experiments, and other Python libraries are used to handle data (jsonlines, json_lines, pandas). Amazon Mechanical Turk was utilized to conduct online studies. The photo editor app on the Samsung Galaxy S21 (version 3.4.2.43) was used to remove objects from the scenes for the generation of scene understanding maps.
Data analysis	Python libraries like numpy, pandas, and scipy were used for analyzing and testing statistical significance, and matplotlib, cv2, and seaborn were used for plotting and visualizing data and images.

For manuscripts utilizing custom algorithms or software that are central to the research but not yet described in published literature, software must be made available to editors and reviewers. We strongly encourage code deposition in a community repository (e.g. GitHub). See the Nature Portfolio guidelines for submitting code & software for further information.

Data

Policy information about availability of data

All manuscripts must include a data availability statement. This statement should provide the following information, where applicable:

- Accession codes, unique identifiers, or web links for publicly available datasets
- A description of any restrictions on data availability
- For clinical datasets or third party data, please ensure that the statement adheres to our policy

The images created as part of the study have been deposited in a Mendeley repository (DOI:https://doi.org/10.17632/z6jb259pcd.1). The repository also contains preprocessed data for plotting the fixation distribution across object categories and the cumulative fixation line plots. The code to access and visualize eye movement data is provided in a GitHub repository (DOI:https://doi.org/10.5281/zenodo.17374055)

Research involving human participants, their data, or biological material

Policy information about studies with human participants or human data. See also policy information about sex, gender (identity/presentation), and sexual orientation and race, ethnicity and racism.

Reporting on sex and gender	Sex and Gender were not considered in the study design. Our main hypothesis is that eyes are directed to regions/objects that maximize scene understanding and are presumed to apply to all genders. We did collect self-reported gender data. Due to a technical problem, we have gender data for 100 out of a total of 260 observers. We had consent to collect the gender data. The data identifies the gender of each subject for which this information was collected.<br>We also collected Amazon Mechanical Turk data but did not have consent and did not collect gender data from those participants. The breakdown of gender for Mechanical Turk participants is reported to be 57% female and 43% male.
Reporting on race, ethnicity, or other socially relevant groupings	Race and ethnicity were not considered in the study design. Our main hypothesis is that eyes are directed to regions/objects that maximize scene understanding and are presumed to apply to all ethnicity. We did collect self-reported ethnicity data, but due to a technical data collection problem, we have gender data for 100 out of a total of 260 observers. We had consent to collect the gender data. The data identifies the ethnicity of each subject for which this information was collected. We also collected Amazon Mechanical Turk data from US workers but did not have consent and did not collect ethnicity data from those participants. The breakdown of ethnicities for Mechanical Turk participants is reported to be 80% Caucasian, 9% African American, 10% Asian, and 1% other. The Hispanics (20%) are represented within the percentages of Caucasian and African American workers.
Population characteristics	See above
Recruitment	The participants were the undergraduate and graduate students of UCSB Psychological Brain Sciences. Participants did not know a priori about the hypothesis or the details of the experiment. Self-selection to participate in the study should not have biased any results. Although the main study was conducted with participants ranging from 18-30 years, we expect the main findings to generalize to other ages.
Ethics oversight	Office of Research and Human Subjects, University of California, Santa Barbara.

Note that full information on the approval of the study protocol must also be provided in the manuscript.

Field-specific reporting

Please select the one below that is the best fit for your research. If you are not sure, read the appropriate sections before making your selection.

☐ Life sciences ☒ Behavioural & social sciences ☐ Ecological, evolutionary & environmental sciences

For a reference copy of the document with all sections, see nature.com/documents/nr-reporting-summary-flat.pdf

Behavioural & social sciences study design

All studies must disclose on these points even when the disclosure is negative.

Study description	This is an eye movement experimental study. Eye movement data is collected on real-world scenes, and we quantitatively analyze the patterns we observe when subjects view scenes using different instructions. It is a between-subjects design.
Research sample	UCSB Psychological and Brain sciences (PBS) undergraduate and graduate population.
Sampling strategy	Participants self-select to sign up for the study from a list of available studies from the UCSB PBS undergraduate. Other participants were randomly assigned to the study. The sample size (n=50 for each condition of the eye movement study was consistent with previous lab eye movement studies with real-world scenes (Koehler & Eckstein, 2014, Journal of Vision; Koehler, Eckstein, Abkas, 2017; Current Biology) and faces (Peterson & Eckstein, 2012) and somewhat smaller than from another lab (n= 79, Henderson & Hayes, 2017). The comparisons of interest motivated by the hypothesis reached statistical significance. We would not expect the sample size to change the findings and conclusions. Also, our statistical analysis critically uses non-parametric bootstrap techniques

	that include variability across subjects and images. This latter source of variability is often ignored in many behavioral studies in psychology.
Data collection	The Winograd images were photographed at the UCSB campus with the help of undergraduate lab research assistants. Identifiable individuals in the images signed a consent that authorized the experimenters to share the images and present them in the public domain. The eye movement data was collected using EyeLink 1000+ desktop-mount eye tracker. No one else was present except the participant while collecting eye movement data for the study. The researcher knew the study's hypothesis but did not share any information about the hypothesis with any participants.
Timing	Start date for data collection: 2022 Jan; End date for data collection: 2025 May.
Data exclusions	We excluded two image pairs from our Winograd dataset because the descriptions provided for those images were variable and vague, and it is of prime importance to have images that people in our experiment consistently describe.
Non-participation	No participants dropped out or declined participation.
Randomization	The allocation of participants to different experimental conditions was random.

Reporting for specific materials, systems and methods

We require information from authors about some types of materials, experimental systems and methods used in many studies. Here, indicate whether each material, system or method listed is relevant to your study. If you are not sure if a list item applies to your research, read the appropriate section before selecting a response.

Materials & experimental systems

n/a	Involved in the study
☒	☐ Antibodies
☒	☐ Eukaryotic cell lines
☒	☐ Palaeontology and archaeology
☒	☐ Animals and other organisms
☒	☐ Clinical data
☒	☐ Dual use research of concern
☒	☐ Plants

Methods

n/a	Involved in the study
☒	☐ ChIP-seq
☒	☐ Flow cytometry
☒	☐ MRI-based neuroimaging

Plants

Seed stocks	*Report on the source of all seed stocks or other plant material used. If applicable, state the seed stock centre and catalogue number. If plant specimens were collected from the field, describe the collection location, date and sampling procedures.*
Novel plant genotypes	*Describe the methods by which all novel plant genotypes were produced. This includes those generated by transgenic approaches, gene editing, chemical/radiation-based mutagenesis and hybridization. For transgenic lines, describe the transformation method, the number of independent lines analyzed and the generation upon which experiments were performed. For gene-edited lines, describe the editor used, the endogenous sequence targeted for editing, the targeting guide RNA sequence (if applicable) and how the editor was applied.*
Authentication	*Describe any authentication procedures for each seed stock used or novel genotype generated. Describe any experiments used to assess the effect of a mutation and, where applicable, how potential secondary effects (e.g. second site T-DNA insertions, mosiacism, off-target gene editing) were examined.*
